# MBNL1-AS1 Promotes Hypoxia-Induced Myocardial Infarction via the miR-132-3p/RAB14/CAMTA1 Axis

**DOI:** 10.1155/2023/3308725

**Published:** 2023-02-04

**Authors:** Yanbing Li, Min Zong, Xiaonan Guan, Xuejiao Wu, Guiling Ma, Yu Wei, Zhi Li

**Affiliations:** ^1^Department of Cardiology, Beijing Youan Hospital, Capital Medical University, Beijing 100069, China; ^2^Department of Cardiology, Beijing Chaoyang Hospital, Capital Medical University, Beijing 100020, China; ^3^Department of Cardiovascular Medicine, the First Affiliated Hospital of Shantou University Medical College, Shantou, 515041 Guangdong, China

## Abstract

**Background:**

Mounting evidence have indicated that long noncoding RNA (lncRNA) muscleblind like splicing regulator 1 antisense RNA 1 (MBNL1-AS1) play a crucial regulatory role in cardiovascular disease, myocardial infarction (MI) included. In this research, we sought to probe into the biological function and potential mechanism of MBNL1-AS1 in MI.

**Methods:**

Cardiomyocytes were treated under hypoxic conditions for 0–12 h. Functional assays including CCK-8 and flow cytometry were performed to assess hypoxia-stimulated cardiomyocyte viability and apoptosis, respectively. Moreover, bioinformatics analysis and mechanical assays were conducted to reveal the competitive endogenous RNA (ceRNA) mechanism of MBNL1-AS1.

**Results:**

The upregulation of MBNL1-AS1 was found in hypoxia-stimulated cardiomyocytes. Functionally, the downregulation of MBNL1-AS1 dramatically promoted hypoxia-induced cardiomyocyte viability and inhibited apoptosis. Mechanistically, miR-132-3p bound to MBNL1-AS1 in hypoxia-induced cardiomyocytes, and miR-132-3p directly targeted RAB14, member RAS oncogene family (RAB14) and calmodulin binding transcription activator 1 (CAMTA1). Furthermore, MBNL1-AS1 upregulates the expression of RAB14 and CAMTA1 in hypoxia-stimulated cardiomyocytes via targeting miR-132-3p.

**Conclusions:**

The current study revealed the critical role of the MBNL1-AS1/miR-132-3p/RAB14/CAMTA1 axis in MI, indicating MBNL1-AS1 as an innovative therapeutic target for MI.

## 1. Introduction

As a kind of ischemic heart disease, myocardial infarction (MI) is accompanied with high morbidity and mortality [1, 2]. Existing medicines and therapeutic methods can only retard the progression of the disease [3, 4]. As a consequence, it is urgent to explore and develop effective treatment strategies to prevent MI.

Noncoding RNAs (ncRNAs), a type of RNAs lack of the potential of protein-coding, were initially recognized as “junk DNAs” [5, 6]. Increasing researches have proved that about 98% of the human genome are ncRNAs, which have modulatory functions and can effectively couple back into a broader communication network [7, 8]. As the two main types of ncRNAs, long ncRNAs (lncRNAs) and microRNAs (miRNAs) have achieved extensive concern. Accumulating evidences have demonstrated that lncRNAs and miRNAs are important regulators in MI. Moreover, lncRNAs may act as competitive endogenous RNAs (ceRNA) to sequester miRNAs in MI development. At the same time, miRNAs usually function in MI through binding to the 3′-untranslated region (3′-UTR) of mRNAs, which affect the translation of mRNAs.

lncRNA MBNL1-AS1 has been widely documented to be implicated in multiple cancers, such as nonsmall cell lung cancer [9], bladder cancer, and retinoblastoma. Given the fact that the functions of MBNL1-AS1 in cancers have been well-studied, some studies have been devoted to exploring the role of MBNL1-AS1 in noncancerous diseases. A study proposed by Li et al. has indicated that MBNL1-AS1 targets KCNMA1 to enhance sevoflurane-pretreated ischemia-reperfusion injury [10]. However, the role of MBNL1-AS1 in MI remains be investigated.

In this research, to reveal the function of MBNL1-AS1 in MI, we constructed an in vitro model of MI in H9c2 cells treated with hypoxia. Moreover, we explored the interaction between MBNL1-AS1 and miRNA as well as the downstream genes. Our study might provide a promising prospect for MI treatment.

## 2. Material and Methods

### 2.1. Cell Culture and Treatment

The rat embryonic ventricular cardiomyocyte H9c2 and human embryonic kidney cell (HEK293T) were procured from American Type Culture Collection. Both H9c2 and HEK293T cells were maintained in DMEM (A4192101, Gibco, Rockville, MD, USA) containing 10% fetal bovine serum with 5% CO_2_ at 37°C. To mimic MI, H9c2 cells were maintained in a hypoxia incubator containing 1% O_2_, 5% CO_2_, and 94% N_2_.

### 2.2. Cell Counting Kit-8 (CCK-8)

Three independent experiments were performed. Cardiomyocytes were cultured with 10 *μ*l CCK-8 solution procured from Dojindo (Gaithersburg, MD, USA) in 96-well plates. The absorbance was measured at 450 nm.

### 2.3. Flow Cytometry

The experiment was performed thrice independently. The FITC-annexin V/PI detection kit procured from Biosea Biotechnology (Beijing, China) was applied as per the user guide. Cardiomyocytes were collected and resuspended in 6-well plates, and then dyed with FITC-annexin V and PI, and assessed by the cytometry procured from Beckman Coulter (Fort Collins, CO, USA).

### 2.4. Western Blot

Three independent experiments were performed. Total protein was extracted via RIPA lysis buffer procured from Santa Cruz Biotechnology (CA, USA). After the proteins were assessed by SDS-PAGE (1610174, Bio-Rad Laboratories, Shanghai, China) and transferred to PVDF (Millipore, MA), 5% nonfat milk (QYR1330, Qualityard, Beijing qualityard biotechnology Co., Ltd.) were applied for membrane blocking. Next, the membranes were incubated with primary antibodies including Bax (Abcam, Cambridge, MA, USA, ab32503), Cleaved caspase-3 (Abcam, ab32042), total caspase-3 (Abcam, ab32351), Bcl-2 (Abcam, ab32124), RAB14 (Invitrogen, PA5-100175), CAMTA1 (Abcam, ab227713), and GAPDH (Abcam, ab181602). After washing, the secondary antibodies procured form Abcam were incubated. The blots were detected using the chemiluminescence detection kit (Pierce, WI, USA).

### 2.5. Quantitative Real-Time Polymerase Chain Reaction (RT-qPCR)

Three experiments were performed independently. Total RNA was extracted from hypoxia-induced cardiomyocytes using the Trizol reagent procured from Life Technologies Corporation (Carlsbad, USA). After the application of First Strand cDNA Synthesis Kit (GeneCopoeia, USA), qPCR was implemented using Takara SYBR® PrimeScript™ PCR kit (11736051, Invitrogen, Carlsbad, CA, USA). Calculation of gene expression was performed using 2^−*ΔΔ*Ct^ method, with GAPDH or U6 as the internal control.

### 2.6. Subcellular Fractionation

Three experiments were performed independently. This assay was carried out via a Nuclear and Cytoplasmic Extraction Reagent (78835, Thermo Fisher Scientific, Rockford, IL, USA). Fractionated RNAs were detected by RT-qPCR analysis.

### 2.7. Fluorescent In Situ Hybridization (FISH)

Three experiments were performed independently. Ribo™ Fluorescent In Situ Hybridization Kit procured from RiboBio (Guangzhou, China) was applied as per the user guide. The MBNL1-AS1 probe was purchased by RiboBio. DAPI (D9542, Sigma-Aldrich, St. Louis, MO, USA) was applied for nuclear staining. Images of both H9c2 and H9c2/Hypoxia cells were captured by confocal microscope (Axio-Imager_LSM-800, Zeiss, Oberkochen, Germany).

### 2.8. Cell Transfection

Three experiments were performed independently. Specific shRNAs for MBNL1-AS1, RAB14, and CAMTA1 were obtained by GenePharma (Shanghai, China) with the sh/NC being negative control. miR-132-3p mimics, miR-132-3p inhibitor, and the negative controls (NC mimics and NC inhibitor) were also supplied by GenePharma. Cell transfection was performed using lipofectamine 3000 reagent (Life Technologies Corporation), and the transfection efficiency was verified by RT-qPCR.

### 2.9. MS2-RNA-Binding Protein Immunoprecipitation (RIP)

Three independent experiments were performed. Maltose-binding protein (MBP)-affinity purification was employed to evaluate miRNAs that are related to MBNL1-AS1. Cells were treated with MS2-tagged MBNL1-AS1 to acquire miRNAs related to MBNL1-AS1. The RIP assay was conducted as previously described [11]. The abundance of miRNAs was measured by RT-qPCR.

### 2.10. Luciferase Reporter Assay

Three experiments were performed independently. The fragment of RAB14 3′-UTR or CAMTA1 3′ UTR or MBNL1-AS1 containing the miR-132-3p binding site were inserted into pmirGLO dual luciferase vector (E1330, Promega, Madison, Wisconsin, USA). The luciferase reporter plasmids were cotransfected into hypoxia-induced cardiomyocytes or HEK293T cells with miR-132-3p mimics. The luciferase activity of wild-type MBNL1-AS1/RAB14/CAMTA1 (MBNL1-AS1-WT, MBNL1-AS1-WT, and CAMTA1-WT) and their mutant types (MBNL1-AS1-MUT, MBNL1-AS1-MUT, and CAMTA1-MUT) was measured by Dual Luciferase Reporter Assay System procured from Promega.

### 2.11. Statistical Analysis

Data of experimental results was presented as mean ± standard deviation and processed by Graphpad Prism 8 software (GraphPad Software, San Diego, United States). Statistical analyses were implemented with SPSS 19.0 software (IBM, Stanford University, United States). Statistical differences were tested by Student's *t* test or one-way analysis of variance (ANOVA). Statistical differences were considered significant when *P* < 0.05.

## 3. Results

### 3.1. Upregulation of MBNL1-AS1 in Hypoxia-Induced H9c2 Cells

**Figure 1:**
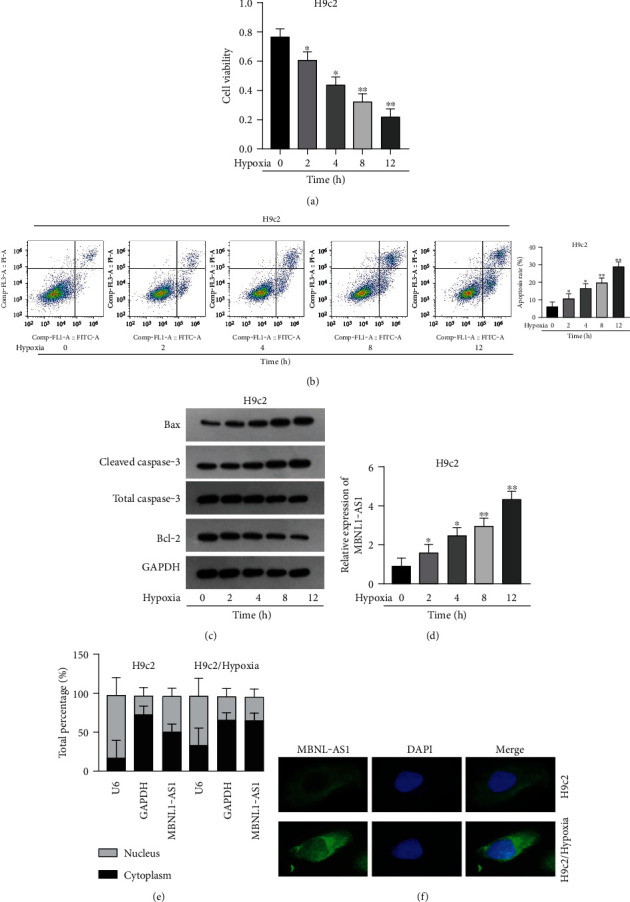
MBNL1-AS1 is highly expressed in hypoxia-induced H9c2 cells. (a) CCK-8 assay examined H9c2 cell viability after treatment of hypoxia for 0–12 h. (b) Flow cytometry analyzed H9c2 cell apoptosis after exposure of hypoxia for 0–12 h. (c) Levels of apoptosis-related proteins were measured in H9c2 cells after hypoxia treatment for 0–12 h via western blot. (d) MBNL1-AS1 expression was examined in H9c2 cells with exposure of hypoxia for 0–12 h by RT-qPCR. (e, f) Cellular location of MBNL1-AS1 was determined by subcellular fractionation and FISH assays. ^∗^*P* < 0.05, ^∗∗^*P* < 0.01.

Firstly, H9c2 cells were subjected to the exposure of hypoxia for 0–12 h, and the viability of H9c2 cells was declined in a time dependent manner (Figure 1(a)). Since cell viability was reduced to about 50% after 8 h of hypoxia treatment, 8 h was chosen as a hypoxia-stimulating condition to be applied in the subsequent assays. Besides, the results from flow cytometry manifested that the apoptosis rate of H9c2 cells was promoted with increasing duration of hypoxia treatment (Figure 1(b)). This phenomenon was further obtained from western blot analysis. As indicated in Figure 1(c), the levels of proapoptotic proteins (Bax and cleaved caspase-3) were significantly elevated whereas that of Bcl-2 (antiapoptotic protein) was decreased as the hypoxia treatment time increased. These data collectively suggested that the MI in vitro model was successfully established. Then, we assessed the expression of MBNL1-AS1 in H9c2 cells subjected to hypoxic treatment using RT-qPCR. The results indicated that MBNL1-AS1 expression was elevated with increasing duration of hypoxia treatment (Figure 1(d)). In addition, the results of subcellular fractionation and FISH assays indicated that MBNL1-AS1 was majorly distributed in the cytoplasm of both H9c2 and hypoxia-induced H9c2 cells (Figures 1(e) and 1(f)), suggesting that MBNL1-AS1 might exert critical functions in hypoxia-induced H9c2 cells via posttranscriptional regulation.

### 3.2. MBNL1-AS1 Depletion Increases the Viability and Decreases the Apoptosis of Hypoxia-Induced H9c2 Cells

**Figure 2:**
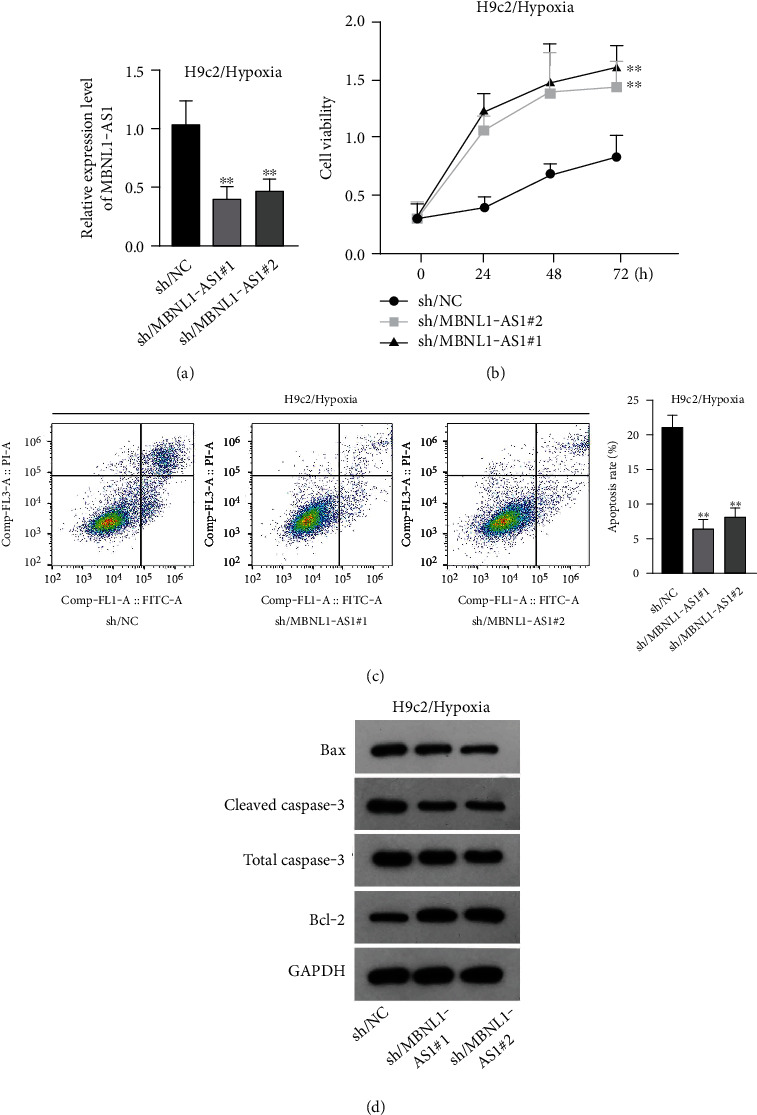
Impacts of MBNL1-AS1 depletion on hypoxia-induced H9c2 cell viability and apoptosis. (a) Silencing efficiency of MBNL1-AS1 was verified by RT-qPCR in hypoxia-induced H9c2 cells. (b) Viability of H9c2 cells after MBNL1-AS1 silencing was examined via CCK-8 assay with 8 h hypoxia treatment. (c) Apoptosis of H9c2 cells after MBNL1-AS1 silencing was examined by flow cytometry analysis, after exposure of hypoxia for 8 h. (d) Levels of apoptosis-related proteins were measured in MBNL1-AS1-depleted H9c2 cells after exposure of hypoxia for 8 h by western blot.^∗∗^*P* < 0.01.

To seek the functional impacts of MBNL1-AS1 in hypoxia-induced H9c2 cells, two specific shRNAs targeting MBNL1-AS1 were transfected into cells to silence MBNL1-AS1 expression. Then, RT-qPCR analysis verified the successful transfection (Figure 2(a)). The results of functional assays revealed that the viability of hypoxia-induced H9c2 cells was increased when MBNL1-AS1 was knocked down (Figure 2(b)). Meanwhile, MBNL1-AS1 depletion diminished the apoptosis rate of H9c2 cells under hypoxia treatment (Figure 2(c)). Additionally, the expression of apoptosis-linked proteins was detected by western blot analysis. The results showed that the expression of Bax and cleaved caspase-3 were decreased in hypoxia-induced H9c2 cells after MBNL1-AS1 deletion but that of Bcl-2 was enhanced (Figure 2(d)).

### 3.3. MBNL1-AS1 Binds to miR-132-3p

**Figure 3:**
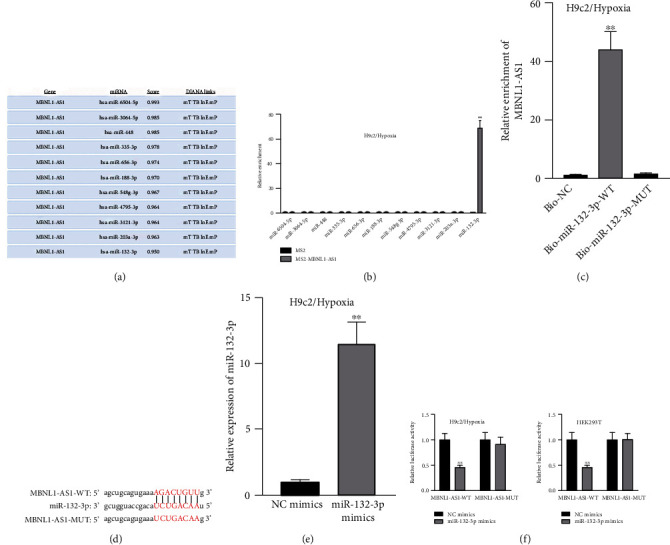
miR-132-3p interacts with MBNL1-AS1. (a) miRNAs bound to MBNL1-AS1 from DIANA database with the threshold value > 0.95. (b) MS2-RIP assay detected the combination between candidate miRNAs and MBNL1-AS1. (c) RNA pull down assay measured the combination between MBNL1-AS1 and miR-132-3p. (d) Binding sites between MBNL1-AS1 and miR-132-3p. (e) Upregulation efficiency of miR-132-3p was measured by RT-qPCR. (f) Luciferase activities of MBNL1-AS1-WT as well as MBNL1-AS1-MUT were detected in hypoxia-treated H9c2 and HEK293T cells after miR-132-3p elevation. ^∗∗^*P* < 0.01.

Given that MBNL1-AS1 was preferentially located in cell cytoplasm, we conjectured that MBNL1-AS1 might work as a ceRNA to sponge specific miRNA in MI progression. Based on DIANA (http://carolina.imis.athena-innovation.gr) tool, we predicted 11 miRNAs harboring binding sites of MBNL1-AS1 with the threshold value > 0.95 (Figure 3(a)). Then MS2-RIP assay was employed to determine the target miRNAs of MBNL1-AS1 in hypoxia-induced H9c2 cells. The results indicated that MS2-tagged MBNL1-AS1 vector was abundant for miR-132-3p (Figure 3(b)). Moreover, the data of RNA pull down assay indicated that MBNL1-AS1 was pulled down by biotin-labeled miR-132-3p wild-type rather than biotin-labeled miR-132-3p mutant-type (Figure 3(c)). In addition, the binding sites between MBNL1-AS1 and miR-132-3p were predicted by starBase (http://starbase.sysu.edu.cn) and presented in Figure 3(d). To further assess the interaction between MBNL1-AS1 and miR-132-3p, we firstly overexpressed miR-132-3p in H9c2/Hypoxia cells (Figure 3(e)). The data of luciferase reporter assay demonstrated that miR-132-3p mimics decreased the luciferase activity of MBNL1-AS1-WT in H9c2/Hypoxia and HEK293T cells, but had no changes on that of MBNL1-AS1-MUT (Figure 3(f)). To validate whether MBNL1-AS1 is involved in MI via interacting with miR-132-3p, RT-qPCR was firstly performed and data manifested that miR-132-3p was downregulated in miR-132-3p inhibitor group than that in the NC group (Figure S1A). Subsequently, rescue assays were carried out. The results indicated that miR-132-3p silencing reversed the increased viability and the reduced apoptosis caused by MBNL1-AS1 depletion in H9c2 cells under hypoxia treatment (Figure S1B-S1C). Similarly, the inhibited protein levels of Bax and cleaved caspase-3 as well as the elevated Bcl-2 levels in MBNL1-AS1-silenced H9c2 cells under hypoxia treatment were restored after cotransfection of miR-132-3p inhibitor (Figure S1D).

### 3.4. miR-132-3p Directly Targets CAMTA1 and RAB14

**Figure 4:**
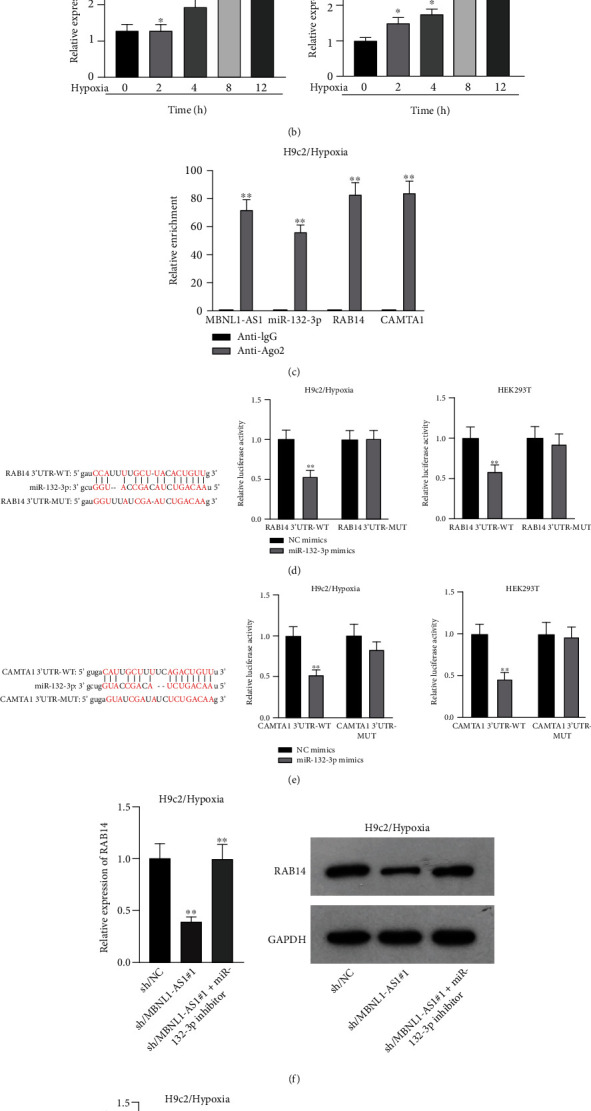
miR-132-3p directly targets CAMTA1 and RAB14. (a) Two mRNAs (CAMTA1 and RAB14) targeted by miR-132-3p were sifted out by four bioinformatics tools including PITA, miRmap, microT, and RNA22. (b) Expression of CAMTA1 and RAB14 were detected in H9c2 cells under hypoxia treatment for 0-12 h. (c) RIP assays detected the abundance of MBNL1-AS1, miR-132-3p, CAMTA1, and RAB14 in anti-Ago2 groups. (d, e) Binding sites between miR-132-3p and RAB14 3′UTR/CAMTA1 3′UTR, and luciferase activities of RAB14 3′UTR-WT/MUT as well as CAMTA1 3′UTR-WT/MUT were assessed in hypoxia-treated H9c2 and HEK293T cells after miR-132-3p overexpression. (f, g) Expression of RAB14 and CAMTA1 were examined in hypoxia-treated H9c2 cells after the transfection of sh/NC, sh/MBNL1-AS1#1, and sh/MBNL1-AS1#1 + miR-132-3p inhibitor, respectively. ^∗∗^*P* < 0.01.

Applying four bioinformatics tools including PITA, miRmap, microT, and RNA22, 2 mRNAs (CAMTA1 and RAB14) targeted by miR-132-3p were sifted out (Figure 4(a)). Then we detected their expression in hypoxia-treated H9c2 cells for 0-12 h. We found that CAMTA1 and RAB14 were upregulated in H9c2 cells in a time-independent manner (Figure 4(b)). Besides, we silenced CAMTA1 and RAB14 expression and found that knockdown of CAMTA1 and RAB14 significantly elevated the viability and inhibited the apoptosis of H9c2 cells under hypoxia treatment (Figure S2A-S2D). To test whether MBNL1-AS1, miR-132-3p, CAMTA1, and RAB14 coexisted in the RNA-induced silencing complex (RISC), RIP assay was performed using Ago2 antibody. It was revealed that these four RNAs were largely abundant in anti-Ago2 groups relative to anti-IgG groups (Figure 4(c)). Moreover, we severally predicted the binding sites of miR-132-3p on RAB14 3′UTR and CAMTA1 3′UTR, and found that miR-132-3p overexpression led to a reduction on the luciferase activities of RAB14 3′UTR-WT and CAMTA1 3′UTR-WT in H9c2/Hypoxia and HEK293T cells, while barely influenced those of RAB14 3′UTR-MUT and CAMTA1 3′UTR-MUT (Figures 4(d) and 4(e)). In addition, we revealed that MBNL1-AS1 silencing markedly declined the expression of RAB14 and CAMTA1, while this effect was counteracted by miR-132-3p silencing (Figures 4(f) and 4(g)).

### 3.5. MBNL1-AS1 Regulates the Viability of Hypoxia-Induced H9c2 Cells via Upregulating RAB14 and CAMTA1

**Figure 5:**
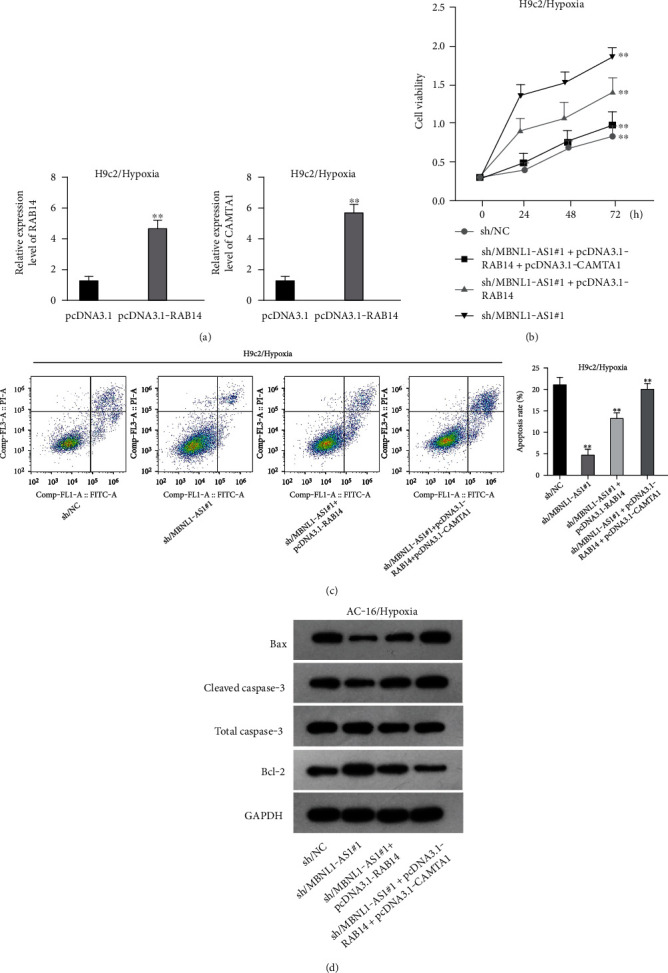
MBNL1-AS1 regulates hypoxia-induced H9c2 cell viability via upregulating RAB14 and CAMTA1. (a) Overexpression efficiency of RAB14 or CAMTA1 was certified by RT-qPCR. Rescue assays were implemented in H9c2 cells after the transfection of sh/NC, sh/MBNL1-AS1#1, sh/MBNL1-AS1#1 + pcDNA3.1-RAB14, and sh/MBNL1-AS1#1 + pcDNA3.1-RAB14 + pcDNA3.1-CAMTA1, respectively. (b) H9c2/Hypoxia cell viability was assessed by CCK-8 assay after different transfections. (c) H9c2/Hypoxia cell apoptosis was assessed through flow cytometry analysis after different transfections. (d) Protein levels of Bax, cleaved caspase-3, and Bcl-2 was measured by western blot. ^∗∗^*P* < 0.01.

We further upregulated RAB14 and CAMTA1 expression (Figure 5(a)), and then separately transfected sh/NC, sh/MBNL1-AS1#1, sh/MBNL1-AS1#1 + pcDNA3.1-RAB14, and sh/MBNL1-AS1#1 + pcDNA3.1-RAB14 + pcDNA3.1-CAMTA1 into hypoxia-treated H9c2 cells for rescue assays. The results indicated that the enhanced cell viability induced by MBNL1-AS1 knockdown under hypoxia treatment was partly offset after RAB14 overexpression, and was fully counteracted after the simultaneous overexpression of RAB14 and CAMTA1 (Figure 5(b)). Besides, the inhibited apoptosis rate in MBNL1-AS1-depleted H9c2 cells under hypoxia treatment was partially abolished when RAB14 was increased together, and was totally counteracted after overexpression of RAB14 and CAMTA1 simultaneously (Figure 5(c)). Likewise, the lessened Bax and cleaved caspase-3 levels as well as the increased Bcl-2 levels in hypoxia-treated H9c2 cells after MBNL1-AS1 downregulation were partly neutralized by RAB14 elevation, and was completely abrogated when RAB14 and CAMTA1 were upregulated together (Figure 5(d)).

## 4. Discussion

A large amount of lncRNAs have been identified to be associated with multiple kinds of cardiovascular diseases, MI included. lncRNA CAIF represses autophagy and improves MI via p53-mediated myocardin transcription [12]. lncRNA H19 inhibits MI-induced myocardial injury through regulating KDM3A [13]. lncRNA Gm2691 impedes apoptosis and inflammatory response after MI via the PI3K/Akt signaling pathway [14]. In this research, we found that lncRNA MBNL1-AS1, a wide coverage gene in cancer progression, was significantly highly expressed in H9c2 cells under hypoxia treatment. Moreover, functional assays proved that MBNL1-AS1 silencing significantly promoted the viability and restrained the apoptosis of hypoxia-induced H9c2 cells. These findings were in line with a previous study about ischemia-reperfusion injury [10], suggesting that lncRNA MBNL1-AS1 might contribute to MI progression.

Recent studies have revealed that lncRNAs interact with miRNAs and exert important functions in the development of diseases. Dysregulation of miRNAs was extensively reported in the progression of MI. miRNA-145 represses MI-induced apoptosis via Akt3/mTOR signaling pathway [15]. miRNA-488-3p impedes acute MI-induced cardiomyocyte apoptosis via modulating ZNF791 [16]. miR-124 influences cardiomyocyte apoptosis and MI via targeting Dhcr24 [17]. Moreover, the lncRNA-miRNA interaction in MI is emerged as an interest topic. In this study, we focused on the relation between MBNL1-AS1 and miRNAs, with the purpose of revealing a potential mechanism of which MBNL1-AS1 promoted MI. We found that MBNL1-AS1 directly bound to miR-132-3p. As reported previously, miR-132-3p is involved in ischemic myocardial injury by interacting with lncRNA TUG1 [18]. In our study, we further showed that MBNL1-AS1 regulates MI in hypoxia-induced H9c2 cells via targeting miR-132-3p. Additionally, miRNAs combines with their target mRNAs to block the mRNA translation. miR-132-3p has been registered to directly target SOX11 to inhibit mantle cell lymphoma progression [19]. In our research, we found that RAB14 and CAMTA1 were targeted by miR-132-3p. Moreover, we validated that MBNL1-AS1 served as a sponge for miR-132-3p, and subsequent regulated RAB14 and CAMTA1 expressions.

## 5. Conclusions

To sum up, we demonstrated that lncRNA MBNL1-AS1 promoted hypoxia-induced MI in H9c2 cells via the regulation of miR-132-3p/RAB14/CAMTA1 axis. Our study might provide an innovative target for the treatment of MI. However, our study also exist some limitations. First, our study did not explore the upregulation mechanism of MBNL1-AS1 in hypoxia-treated H9c2 cells. Moreover, our study did not investigate whether MBNL1-AS1 is involved in MI via the modulation of certain pathways. All of these deficiencies will be improved in the near future.

## Data Availability

The data used to support the findings of this study are included within the article.

## Abbreviations

3′-UTR: 3′-Untranslated region
ANOVA: Analysis of variance
CAMTA1: Calmodulin binding transcription activator 1
CCK-8: Cell counting kit-8
ceRNA: Competitive endogenous RNA
FISH: Fluorescent in situ hybridization
lncRNA: Long noncoding RNA
MBP: Maltose-binding protein
miRNAs: MicroRNAs
MBNL1-AS1: Muscleblind like splicing regulator 1 antisense RNA 1
MI: Myocardial infarction
ncRNAs: Noncoding RNAs
RT-qPCR: Quantitative real-time polymerase chain reaction
RAB14: RAB14, member RAS oncogene family
RIP: RNA-binding protein immunoprecipitation
RISC: RNA-induced silencing complex.

## Supplementary Materials

Supplementary Materials Figure S1: MBNL1-AS1 regulates the viability and apoptosis in hypoxia-induced H9c2 cells via miR-132-3p. Figure S2: knockdown of RAB14 and CAMTA1 increase the viability and decrease the apoptosis in hypoxia-induced H9c2 cells.
