# Supplementary material for: MBNL1-AS1 Promotes Hypoxia-Induced Myocardial Infarction via the miR-132-3p/RAB14/CAMTA1 Axis
Source: Oxid Med Cell Longev. 2023 Feb 4;2023:3308725. doi: 10.1155/2023/3308725 (PMC9922191; doi:10.1155/2023/3308725)

**Figure S1 MBNL1-AS1 regulates the viability and apoptosis in hypoxia-induced H9c2 cells via miR-132-3p.** A Overexpression efficiency of miR-132-3p was certified by RT-qPCR. Rescue assays were performed in H9c2 cells transfected with sh/NC, sh/MBNL1-AS1#1 and sh/MBNL1-AS1#1+miR-132-3p inhibitor, respectively. B Viability from CCK-8 assay. C Apoptosis from flow cytometry analysis. D Protein levels of Bax, cleaved caspase-3 and Bcl-2 from western blot. ^**^P<0.01.


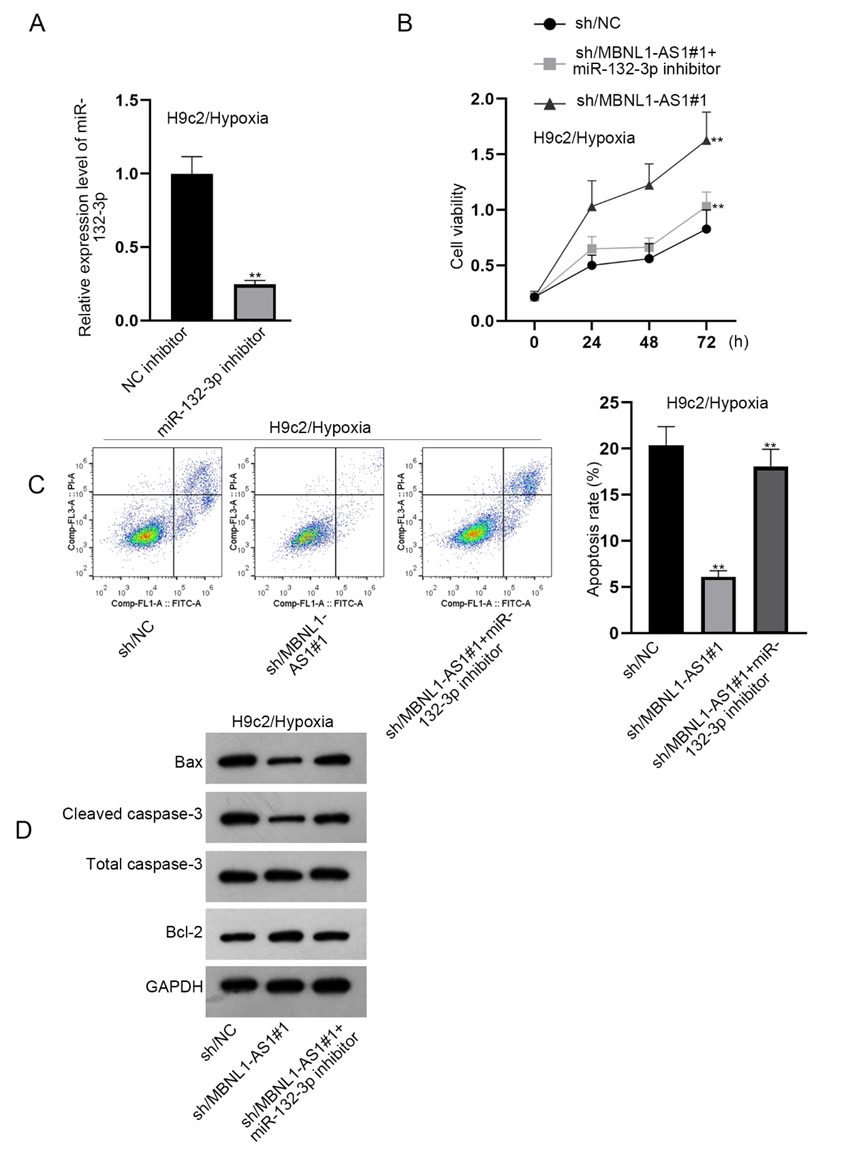


**Figure S2 Knockdown of RAB14 and CAMTA1 increase the viability and decrease the apoptosis in hypoxia-induced H9c2 cells.** A Silencing efficiency of RAB14 or CAMTA1 in hypoxia-induced H9c2 cells was verified by RT-qPCR. B H9c2 cell viability after RAB14 or CAMTA1 silence was examined by CCK-8 assay, after exposure of hypoxia for 8 h. C Apoptosis of H9c2 cells after RAB14 or CAMTA1 silence was examined by flow cytometry analysis, after exposure of hypoxia for 8 h. D Protein levels of Bax, cleaved caspase-3 and Bcl-2 were measured in RAB14- or CAMTA1- depleted H9c2 cells after exposure of hypoxia for 8 h by western blot. ^**^P<0.01.


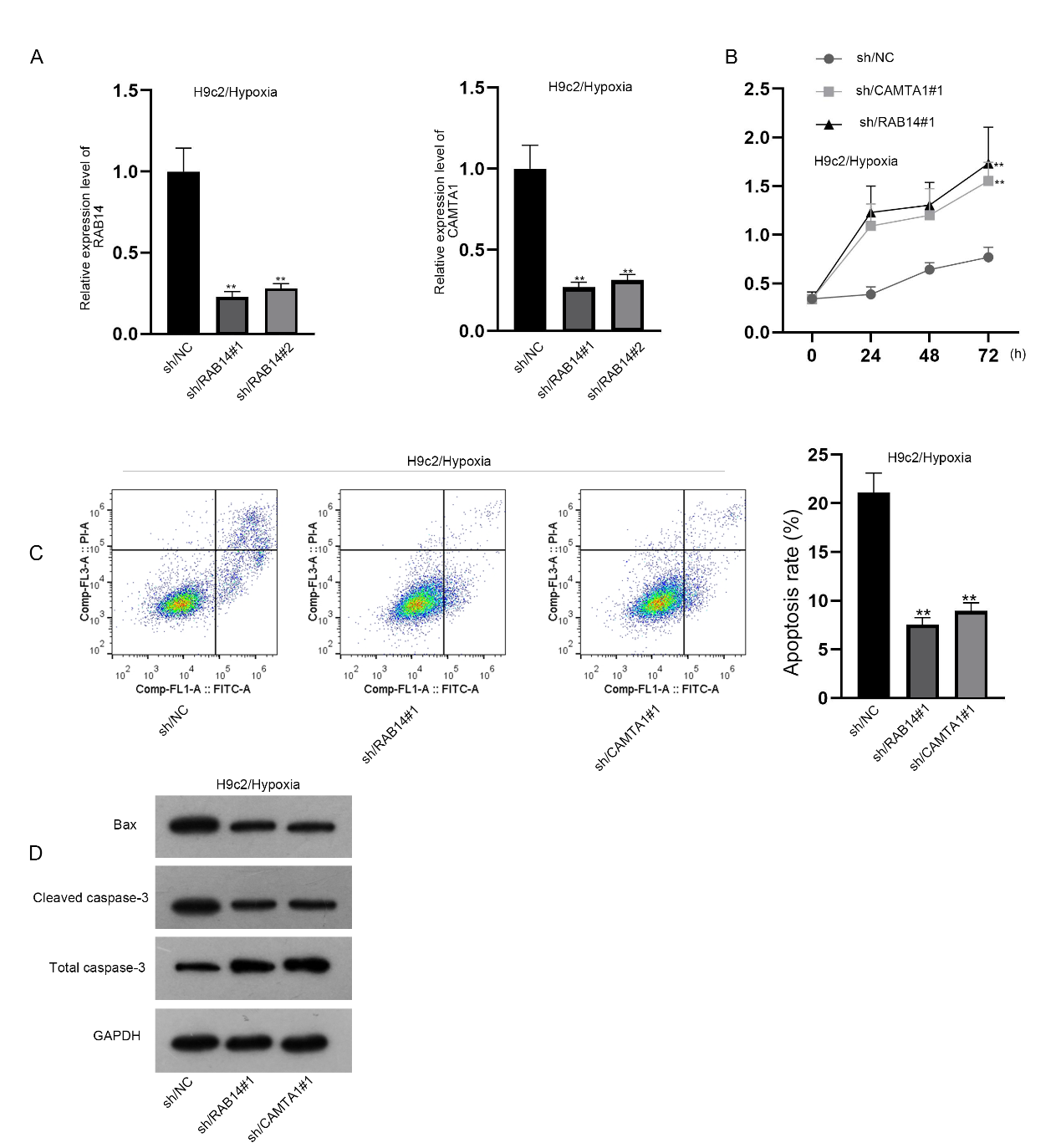

Supplement: Supplementary Materials — Figure S1: MBNL1-AS1 regulates the viability and apoptosis in hypoxia-induced H9c2 cells via miR-132-3p. Figure S2: knockdown of RAB14 and CAMTA1 increase the viability and decrease the apoptosis in hypoxia-induced H9c2 cells. [file 3308725.f1.docx]
